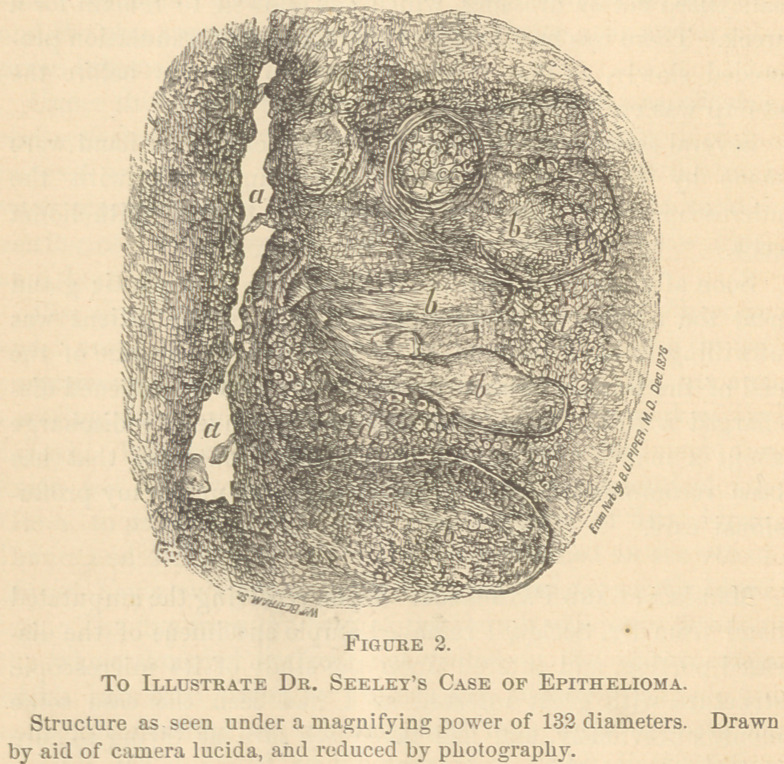# Pathological Transactions of the Chicago Medical Society

**Published:** 1877-01

**Authors:** 


### THE PATHOLOGICAL TRANSACTIONS OF THE CHICAGO MEDICAL SOCIETY.

EDITED BY DR. I. N. DANFORTH.

I.

### CASE OF EPITHELIOMA.

BY T. P. SEELEY, A.M., M.D.

Read before the Chicago Medical Society, Nov. 6, 1876.

W. H., American, male, aged seventy, a hotel proprietor, tall and spare, called at my office January 10, 1876. He was suffering from an extensive ulcer of the palmar surface of the right thumb, extending from the middle of the hand nearly to the second joint, the first joint being much inflamed.

The history of the case, as given by the patient, was that fifty years ago, at the age of twenty, while leaning on an old fashioned mantel piece, he fainted, fell into the fire and burned the hand so extensively that all the fingers had to be amputated through the first phalanges. The other arm and the back were also severely burned. It was about a year before the ulcer entirely healed, and frequently since then it has become fissured and sore, especially in the winter time.

A year ago last winter it broke out again, and the treatment by plasters and ointment, by which before it had been healed, entirely failed.

In December last a homœopathist applied a solution of chloride of zinc and tincture of blood root for twenty-four hours, producing intense pain and increasing the size of the ulcer. A second application for thirty-six hours produced such agony that the patient could endure it no longer; hence he removed the application, finding the first joint inflamed and the ulcer still more extended.

It was soon after this that he came to me. The granulations were excessive and weak, and he was suffering so much pain that he had little rest day or night, and very little appetite. Anodyne lotions, on patent lint, were first applied, and the arm supported in a sling, and quinine, iron and opium given internally.

Under this treatment the acute inflammation was allayed, the suffering much relieved and healthy granulations commenced around the edges. The central fungus granulations were repressed by nitric acid. Nitrate of silver and various astringents were applied, and support, and a moderate pressure kept up by adhesive straps.

The cicatrization, however, did not proceed satisfactorily, and I proposed the operation of removing the thumb at the carpo-metacarpal joint with the excessive granulations, and covering the ulcer with the integuments from the back of the thumb. The patient was very much opposed to the use of the knife, but was at length persuaded, on the approval of Dr. Gunn, to submit to it. With the assistance of Drs. Fitch, Brauer and Bridge, the operation was performed on the 9th of March, Esmarch's apparatus being used and very little blood lost. The flap from the back of the thumb was made to cover the whole denuded surface, and united to the palmar edge which had been pared, by sutures and adhesive straps. An antiseptic dressing of cotton soaked in a weak solution of salicylic acid was used, on the removal of which, in about five days, the flap was found united to the surface, and a portion of the border, leaving about one-half to close by granulation. This process went on rather slowly until the patient, about a month after the operation, took a severe cold from exposure to a north wind on returning from my office. He had a decided chill followed by acute bronchitis. Fearing pyæmia, I made a liberal use of quinine and iron, under which treatment he gradually improved, but the portion of the edge of the flap which was not united began to deteriorate, and, notwithstanding all that could be done, including the use, for a week, of boracic acid, suggested by Dr. Gunn, the ulcer increased slowly until the sixth of June, when, with the assistance of Drs. Graham and VanBuren, I amputated the forearm a little above the middle to avoid one of the old cicatrices. With the exception of the skin the parts united well under the salicylized cotton dressing, which was allowed to remain for a week. There was hardly any suppuration, but granulation proceeded slowly, so that it was about two months before the stump was entirely healed.

Several sections of the border of the ulcer in the hand were made by Dr. Danforth, which, on examination with the microscope, were found to contain the typical epithelioma cells.

Soon after the cicatrization of the stump a lymphatic gland near the axilla was found enlarged; and as the patient was unwilling to have it removed, it increased to the size of the end of the thumb, became inflamed, and on being opened discharged a thick, cheesy pus, and has continued to discharge about a month, the patient using some ointment that has been recommended to him, not being now under my professional care.

Microscopy and Pathology. Upon receiving the amputated hand from Dr. Seeley, I removed ample specimens of the diseased portion, and entered upon the study of its microscopic structure with great interest, as I had seen the case some months previously, and had taken *my* turn at trying to cure it. Numerous thin sections were made in various directions. These were then stained with Beale's carmine, slowly dehydrated by alcohol, passed through oil of cloves to remove the alcohol, and mounted in Woodward's solution of balsam in benzole. Upon carefully examining the sections under different magnifying powers, I could come to but one conclusion, namely: that the case was one of undoubted and undeniable epithelioma. The typical structure of skin-cancer was too manifest to admit of a doubt. The so-called " cancer cylinders " were present in great perfection, and as the thin slices were made in various directions I could study them from different aspects. Out of a considerable number of mounted sections I selected the one which seemed to me, in the most perfect manner, to illustrate the history and probable mode of development of epithelioma in the present instance.

FIGURE 2.

TO ILLUSTRATE DR. SEELEY'S CASE OF EPITHELIOMA.

Structure as seen under a magnifying power of 132 diameters. Drawn by aid of camera lucida, and reduced by photography.

Fig. 2 shows, with very great accuracy, the appearance presented by a section cut in the direction of the hair follicles and sebaceous and sweat glands; that is, through the diseased tissue perpendicularly, or at a right angle to the exposed surface. The open space at *a* is probably a hair follicle divided longitudinally. It is much distorted by the changes in the surrounding connective tissue, and when first examined was distended by an accumulation of large polygonal nucleated cells, due to the atypical proliferation of the compound endothelial layer of the follicle.

Subsequently, by the process of dehydration, and the various manipulations incident to "mounting" the specimen, these cells were mostly washed away; a few of them are seen, however, in the figure. It is possible that, instead of being a true hair follicle, the space referred may have been one of the subdivisions of a sebaceous gland, opening into a hair follicle; but I judged it to be the latter, because, after the section was made, a fragment of a diseased hair remained in the space, and could be distinctly seen, both by myself and others who examined the specimen.

Of course, however, it makes no practical difference which of these two glandular structures it may have been. The real point is as to the epithelial or non-epithelial origin of the new cell growth. At *b b b* are seen several of the so-called "cancer cylinders," divided longitudinally, and these are very perfectly shown, both in the specimen and in the figure. These cylindriform bodies, when first examined in glycerine, were found to be literally stuffed with cells which closely resemble those in the open space already described. But they are rendered so exceedingly transparent by the action of oil of cloves and balsam that they can scarcely be seen by the camera lucida; hence, there are but comparatively few shown in the figure. They are very plainly demonstrable under the microscope, by using a Hartnack No. 5 obj. with a somewhat dim oblique illumination. These "cylinders" are manifestly the product of the rapid and lawless multiplication of the epithelial cells of the degenerated hair follicle. The resemblance between the cells of each is so close as to at once suggest a common origin, and there can be no doubt that this conclusion is warranted by an examination of the section. They are in fact true epithelial cells; the demoralized and degenerated children of those cells which originally formed the inner and outer "root sheaths" of the hair follicle. But they are growing in a direction contrary to that which their law of growth commands. Their physiological growth is always *toward* the surface of the body; in the present instance they are obstinately pushing their way downwards into the connective tissue.

In reference to the development of cylinders of epithelial cancer, Mr. Arnott remarks: "In fact, the new growth seems to consist simply of masses of surface epithelium; which, instead of appearing above and between the papillæ, dip down amongst the connective tissue, and there actively multiplying and thriving as much from the unwonted supply of fluid nourishment as from the absence of the desiccating process to which they are normally subjected as they are pushed on toward the surface of the body, form large tubular and branching collections, capable of more prolific development the further they are removed from the surface, and at the same time more freely subjected to the risk of single cells being taken up and hurried away in the lymph or blood streams to form similar collections elsewhere." ("Cancer: its Varieties, etc." By Henry Arnott, F.R.C.S., p. 69.) It would, perhaps, be difficult to get the essential facts concerning the pathology of epithelioma into fewer words.

As shown in the figure, each cylinder seems to possess a distinct membranous wall; and this wall seems to be a process pushed out from the hair follicle by the distending force of the growing cells contained within them. At a later stage these walls probably undergo degeneration, and their cellular contents are consequently liberated; at this period the danger of blood or lymph infection, and consequently of secondary centers of growth, would begin.

At *c* is shown one of the cylinders which happened to be cut transversely; possibly it is a section near the club-shaped extremity or bottom of a cylinder which proceeded from a neighboring hair follicle or sebaceous gland. The thickness of the wall of the cylinder is well shown. It is also seen to be crowded with large nucleated cells resembling epithelial cells. Although not in accordance with the general teachings upon the subject, I am inclined to believe that the "pearly globules" or "bird's nest bodies" ("*globes épidermiques*" of French authors) are sometimes produced by the concentric development of successive layers of cells, and their imprisonment within the cylinder wall. Intense compression would ensue, the well known tendency of epidermic cells to dry up and become horny would naturally follow, and thus the pearly globule would result from merely mechanical causes.

The letters *d d* refer to the "small celled brood" of Woodward, the "small celled infiltration" of Arnott. The origin of these inter-cylindrical groups of small cells is one of the open questions of our present pathology. Whether they are exclusively the product of a rapid proliferation of the connective tissue — "the invading epithelial growth being preceded by a small celled infiltration, suggesting irritative hyperplasia of the connective tissue nuclei."—(Arnott); or whether they are exclusively the product of the migrating leucocytes and their progeny; or, lastly, whether they are partly contributed by both these sources, are questions which are not yet decided authoritatively. I am inclined to believe that the latter proposition will prove eventually to be the true one. But, however this may be, it is certain that the inter-cylindrical spaces of growing epitheliomata are always filled by a luxuriant growth of cells, considerably smaller than those which form the cylinders; and this small celled brood generally precedes the growth of the cylinders. These cell-groups are doubtless due to an "irritative hyperplasia" of something. In my own view at the present time, they are the evidence and outcome of a reversion to the amœboid type of growth, both of the wandering leucocytes and the nuclei of the connective tissue corpuscles.

I have dwelt upon the histology of this case at some length because I regard it as one of great interest pathologically. It seems to be a case of *induced* epithelioma, quite local in its character in its earlier stages. I think a brief review of its history will lead us to this conclusion: Fifty years previously the tissues involved were badly damaged and their nutrition seriously and permanently interfered with by a severe burn.

In consequence of this, progressive contraction of the cicatrix had taken place, and a still greater interruption of nutrition had resulted. During all these fifty years it has frequently become "fissured and sore, especially in winter time." In the winter of 1874 "it broke out again," and the usual treatment (probably owing to advancing age, and the consequent feebler nutritive capacity of the scar-tissue) failed to produce the usual beneficial effects. Then the patient commenced a series of wanderings from doctor to doctor, and the number of remedies applied almost equalled the possibilities of the Pharmacopœia. In August, 1875, he consulted me. The ulcer had then assumed the appearance of incipient cancroid, and I advised an operation, which disgusted him and he resumed his wanderings. In December following the barbarism of applying chloride of zinc and blood-root was committed, and prior to this electricity had been employed. In fact, the simple ulcer which formed upon the surface of a cicatrix fifty years old, upon the palmar surface of the thumb of a man seventy years old, was persistently teased and worried by the application of irritating remedies, aided somewhat perhaps by the well known disposition of the patient to examine the sore too often and renew the dressing too frequently, into degenerating from an innocent affair to an epithelial cancer. And this, I am persuaded, is a not very uncommon cause of the origin of the epitheliomata.

I. N. D.

Reported Nov. 6, 1876.

The editor of the "Transactions" desires to express his obligations to Dr. R. U. Piper for the beautiful camera drawing from which the foregoing illustration was made; and also to Mr. Bertram for the skill and fidelity with which he has reproduced Dr. Piper's drawing by the process of photographing on wood. It is proper also to remind the readers of the "Transactions" that such illustrations are somewhat costly, and that they are under obligations to the enterprising publishers, Messrs. W. B. Keen, Cooke & Co., who have kindly consented to incur the necessary expenditure.

### II.

### CEREBRAL EMBOLISM AND THROMBOSIS.

Four Cases, Reported by Prof. HENRY M. LYMAN, M.D.

During the past summer I have been so fortunate as to see the *post mortem* appearances in four cases of obstructed cerebral circulation. Three of these cases were interesting because they serve to illustrate the cause of alarming symptoms and sudden death, where, under ordinary circumstances, recovery, or, at least, considerable duration of life, might have been expected. The fourth presented only what is liable to occur in any case of lingering death, with great depression of cardiac vigor and a fibrinous condition of the blood.

Case I. N., a middle aged Irish laborer, was admitted to the County Hospital last May. He was suffering severe pain in the right thoracic region, and there was a small effusion into the right pleural cavity. The pain soon manifested itself in the right shoulder, causing great distress on motion of the joint. Subdued in this region, the pain declared itself over the heart, and a loud mitral murmur became audible. About a week after this the patient became suddenly hemiplegic — waking up in the night and finding himself unable to move his left side. He was still conscious and could articulate tolerably well when I saw him the next day, twelve hours after the accident. The diagnosis was, obstruction of the right middle cerebral artery by a vegetation detached from the mitral valve. The patient was utterly unable to move the left side, and gradually sunk into a state of speechlessness and insensibility. Death occurred on the third day.

At the autopsy about a pint of slightly turbid serum was found in the right thoracic cavity. The mitral valve was covered with recent exudation. At the bifurcation of the right middle cerebral artery was found a solid fibrinous mass, as large as a grain of wheat, completely obstructing the vessel and arresting the passage of the blood. It is worthy of remark that the left side of the brain is more frequently than the right side reached by such embolic visitors from the heart.

Case II. M., aged eighteen, a domestic, of American birth, entered the hospital July 12, 1876. She was well known in the house, having been previously treated there for epilepsy, a disease with which she had been afflicted from childhood. She had several paroxysms of convulsions during the fortnight which followed her admission, but in other respects she seemed to be quite well.

One morning, however, two weeks after coming into the hospital, she was attacked with violent vomiting and diarrhœa. She complained also of a sore throat and of a high fever. Next morning she was covered with the rash of scarlatina. The vomiting and diarrhœa did not cease. She complained of severe pain in the precordial region, and was evidently much prostrated. That evening she had a convulsion, and from that time she passed from one convulsion into another until she died, about two o'clock the next morning.

At the autopsy, twelve hours after death, the skin was still brilliantly injected by the eruption. There was also a vivid arborescent injection of the entire peritoneal and pleural surfaces. The interior of the pericardial sac presented a striking contrast by its normal appearance. The mucous surface of the stomach and intestines did not present any morbid appearances to the naked eye. The liver was healthy. The kidneys were intensely congested, but were not degenerated. The lungs and air passages, as high as the vocal chords, were models of healthy tissue. The mucous surface of the œsophagus from the stomach to the level of the glottis was pale and normal. From that level the entire lining of the pharnyx and posterior portion of the mouth was colored with a dark purple hue. There was little if any moisture in the serous cavities of the body.

On opening the cranium an intense hyperæmia was manifested. The superficial cerebral veins were enormously distended. This condition was the result of an occlusion of the superior longitudinal sinus by a firm, straw-colored clot, exactly like the white heart clots which are so frequently displayed upon the autopsy table. The left lateral sinus was filled with coagulated blood, which had not lost its color. A very small black coagulum was found in the left ventricle. The blood in other parts of the body was fluid.

In this case the local arrest of circulation doubtless served to divert the principal volume of the blood current, through extraordinary channels, upon the ganglia at the base of the brain. Already modified in their structure by previous disease, this unnatural hyperæmia served to exalt their function to such a pathological degree that excessive vomiting, diarrhœa, resulting from vaso-motor disturbance, and, finally, convulsions were excited, and the patient speedily succumbed to the violence of the commotion thus aroused.

Case III. S., a vigorous, middle aged gentleman, was one day so unfortunate as swallow a fragment of chicken bone, which was arrested in its progress just within the *sphincter ani.* At that point it had excited considerable ulceration before it was discovered and removed. The ulcer did not heal well. The superficial fibres of the sphincter were incised by one physician, another did something else. The patient traveled from one doctor to another until it could no longer be denied that he was the victim of a cancer of the rectum. He finally came under the care of Prof. Edwin Powell, M.D., by whom he was attended till death put an end to his suffering. Through the kindness of Prof. Powell I was permitted to witness the autopsy, which was performed mainly for the purpose of ascertaining the cause of certain cerebral symptoms which were manifested during the few days which preceded dissolution.

The patient, who had previously exhibited no cerebral disorder, was attacked with excruciating pain in the head. After a variable period of anguish he would become insensible, would continue for a time in a state of stupor; from which he would gradually emerge, only to renew his experience of pain, succeeded again by unconsciousness. In this way the last week of his life was passed.

At the autopsy we discovered extensive scirrhus cancer of the walls of the rectum. Small nodules of cancerous deposit were appearing upon the omentum. The other viscera presented nothing worthy of extended remark. On opening the head the greater portion of the cerebral convolutions appeared unusually pale. The sulci were filled with serous fluid, especially upon the left side of the brain, where a vein which occupied the posterior branch of the sylvian fissure was filled with black coagulum as far as its junction with the lateral sinus. This seemed to account for the symptoms which appeared before death. Diverted in considerable part from its normal course, by the occlusion of so important a channel, the blood was poured with too voluminous a current upon certain portions of the brain, in which it produced a hyperæmia with sensations of pain until the unwonted pressure was sufficient to occasion insensibility. A period of diminished circulation would then succeed, and the diminution of pressure thus produced would permit the resumption of cerebral function and the return of consciousness. Renewed activity would excite a renewed afflux of blood with consequent renewal of pain, and so forth, until complete exhaustion closed the scene.

CASE IV. Opening the cranium of an emaciated patient who died of pulmonary consumption, I found the superior longitudinal sinus occupied by a slender, straw-colored clot. There was no engorgement of the cerebral veins, nor had there been any cerebral symptoms during the last hours of life

Reported Nov. 20, 1876.

III.

### HYDATIDIFORM DEGENERATION OF THE CHORION.

BY CHAS. WARRINGTON EARLE, M.D.

During January, of the present year, I was consulted by one of my patients, who gave me the following facts in regard to her case:

She became pregnant about December 1, 1875, and five weeks after, a lady acquaintance attempted to produce an abortion by inserting a pointed ivory instrument into the cavity of the uterus. Only a slight discharge of blood followed this operation. Her breasts had continued to enlarge, and the morning sickness had been very severe ever since. No digital examination was permitted, and the lady departed with a few bismuth powders to allay the gastric irritability.

Early in February I visited my patient and found that a day or two since a watery sanguineous discharge had commenced, which at times was very copious and then disappeared for a few hours. A digital examination revealed the os soft and the uterus enlarged.

As the case progressed the watery discharges continued; the abdomen and mammæ enlarged, the uterus seeming to extend more laterally than upward. The patient's nutrition was exceedingly poor, and an anæmic condition speedily ensued. About March 1st, possibly the 4th, a mass described as large as half a hand, slightly rounded and covered with blood-vessels, having a little membranous string attached, was discharged from the vagina. I did not see this product, but give the description as it was given to me by the ladies who were present. The watery discharges, with occasionally a little blood, continued, and there was no diminution in the size of the abdomen. March 12th, the patient suffered more than usual, and at the suggestion of a friend took her bed and applied a hot hop poultice to the abdomen. A short time after pains commenced, and suddenly with a gush, nearly, if not quite, a quart of hydatid like bodies came from the vagina. The lady and friends were now greatly excited, and several physicians were summoned during the following hour, at which time I arrived. The pulse was 150, and considerable blood was being lost. She was ordered Squibb's fl. ex. ergot, and wine when necessary. During the night quite a number of bladder like bodies came away, but these with the hemorrhage gradually ceased, and with a tonic of iron and quinine she made a good recovery by the 20th of the month.

The above case, with the following notes from the literature on the subject, was presented to the Chicago Medical Society, at its annual meeting, April 3d:

I. Substances, generally substantial in texture, the exact nature of which is not always easy to determine, are frequently discharged from the uterus. When they take place independent of impregnation they are called *false moles.* The *true*

*mole* is always the result of conception. In the early days of medicine most fabulous reports were believed in regard to these productions, and judging from the reputed expressions of some so-called medical men and numerous of the laity, there is still a taint of these old ideas remaining in the minds of some.

II. Substances, cyst-like in their appearance, are occasionally discharged from the uterus. It has been supposed until recently that these bladder-like bodies were *true hydatids*, and due to the acephalocyst. Recent microscopical investigations, however, prove that they are entirely different, both in their general formation and in their histological arrangement. A true hydatid of the uterus is made up of closed sacs within other closed sacs, and must contain echinococci heads, etc., etc. True uterine hydatids are very rare. Rokitansky has seen one case, and Graily Hewitt, in Vol. xii, Obst. Trans., reports a second case.

III. The hydatidiform mole, the name now given to the vesicular bodies which most frequently escape from the uterus, are simply little bladders or cysts, not inclosing other cysts, but filled with a watery albuminoid material, and furnishing no evidence whatever of hydatid origin. These vesicles, under the influence of perverted nutrition or development, spring from the villi of the chorion, and may vary in size from a walnut to a millet seed. The cause of this chorionic degeneration is not well understood. Some authorities believe the change takes place previous to the death of the embryo from some form of malformation, while others urge that an accident to or death of the embryo, diverts the developing force from its proper channel, and the degeneration commences.

The pathological specimen was submitted to Dr. Norman Bridge, for microscopical examination, who informs me that no evidence of the echinococcus could be found.

I have not been able to find, in the literature at my command, any case reported where this vesicular degeneration has occurred a second time in the same patient; indeed, I am of the opinion that somewhere in my reading, although I can not now find the article, it is stated that it does not take place the second time. However, it did take place a second time in my patient, although to a less extent than at first.

Aug. 17. Was called in haste to the same lady, whom I found suffering from great hemorrhage; she having lost a short time before I arrived more than half a vessel of blood. In this fluid I found a number of cystic bodies, some of them the size of a grape. Only a few were passed, compared with the former amount. A good recovery was made in ten days, and excellent health has been enjoyed since.

I should add that after the first accident a few applications were made to the uterine mucous membrane, such applicants as the tr. ferri chl. being used, and continued as long as any diseased condition could be perceived.

In regard to the second accident, I suppose pregnancy had again taken place, abortion following quickly from the cystic degeneration of a chorion not yet quite healthly enough to perform normal function.

Reported April 3, 1876.

### IV.

### TUBERCULOSIS IN THE LUNGS OF A CANARY BIRD.

By DR. I. N. DANFORTH,

A few days ago a lady consulted me in regard to her pet canary's health. The "patient" hung his harp on the willows quite a long time previously, appeared languid and drooping, sitting sometimes for an hour or more upon his perch without change of position; took but little food, and, in fact, according to the description given to me, "appeared to be sick all over."

Shortly after this the bird died, and I obtained permission to make a *post mortem* examination. The emaciation of the body was extreme, the plumage was scant and shabby, and all appearances indicated very marked mal-nutrition. Upon opening the body I found an extensive deposit in both lungs which strongly reminded me of ordinary tubercle. Upon microscopic examination of a specimen of this deposit I found that it was indeed tubercular; the usual shrivelled, pinched and degenerate cells appearing in great numbers. No other evidences of disease were noticed. The bird therefore must have died of true tubercular consumption; and it is interesting to notice that the history and progress of the bird's illness was quite typical of this disease.

Although it is well known that tuberculosis is a not very uncommon disease among the lower animals, it is probable that it is much more common than we have supposed. This is especally true of tropical animals brought into our climate and kept in confinement. I well remember the case of a lion who died while some menagerie, to which he belonged, was being exhibited here. His remains were bequeathed to Rush Medical College, and a *post mortem* was made. The lungs were found to be loaded with tubercle in all stages of development and decline.

Caged monkeys are likewise notoriously liable to tuberculosis, and the same fact has been observed with respect to rabbits closely confined in filthy quarters.

The case detailed interests us only as it relates to the general subject of the efficient causes of tuberculosis. The canary bird was kept for the most part in a damp, dark basement, and was consequenly on a short allowance of fresh air and sunlight, both of which are indispensable to healthful bird-life. I have very good reasons for believing also that insufficient care and a want of proper food supply, as to quality and variety, were in part responsible for the degenerated type of cell-life which invaded the lungs of the little songster.

In a word, a canary bird is placed under conditions favorable to the development of tuberculosis, and a tuberculous deposit promptly takes place in the lungs. This seems to show that even birds enjoy no immunity from tuberculous disease when they are forced into situations which invite the genesis of tubercle. In view of the experimental results as to the inocculability of tuberculosis, it is possible that the liability of birds to this disease—if they are generally so liable—may yet come to have a very practical bearing in its relations to their consumption as food by the human family.

Reported Nov. 13, 1876.

## Figures and Tables

**Figure 2. f1:**